# Self-handicapping strategies in educational context: construction and validation of the Brazilian Self-Handicapping Strategies Scale (EEAPREJ)

**DOI:** 10.1186/s41155-022-00210-6

**Published:** 2022-04-02

**Authors:** Evely Boruchovitch, Sueli Edi Rufini, Danielle Ribeiro Ganda, Lucia Cerqueira Miranda, Leandro Silva de Almeida

**Affiliations:** 1 Universidade Estadual de Campinas, Campinas, Brazil; 2 Universidade Estadual de Londrina, Londrina, Brazil; 3 Prefeitura Municipal de Uberlândia - Setor: Educação, Uberlândia, Brazil; 4 Centro de Investigación en Psicopedagogía e Investigaciones, Psicopedagógicas, CIPsp, Buenos Aires, Argentina; 5 Universidade do Minho, Braga, Portugal

**Keywords:** Self-handicap, Self-handicapping strategies, Assessment measures, Higher education

## Abstract

Self-handicapping strategies refer to the set of choices and attitudes adopted to minimize blame for failure and increase the value of success in achievement situations. This paper aims to describe the stages of construction and the psychometric analysis of a scale to measure the self-handicapping strategies of university students. In study 1, the major steps for the construction of the scales and initial results are reported. The internal consistency indices were acceptable and the principal component analysis revealed factors with little explanation of data variance. In study 2, data from a sample of 834 students from several undergraduate courses of different Brazilian universities were subjected to exploratory factor analysis using the minimum rank factor analysis (MRFA) method and the matrix of polychoric correlations. The parallel analysis criterion for factor retention indicated the one-factor solution as the best fit to data. The importance of having a valid and reliable measure to assess self-handicapping strategies in educational contexts and the promising use of the scale in actions to improve learning in higher education are discussed.

## Introduction

Situations that force students to publicly expose their skills and abilities are commonly seen in academic context. The presence of professors and other students as observers in such environments may lead some students fear the failure or doubt about their abilities. In this case, students may use self-handicapping strategies, which are actual or fictitious obstacles or behavioral or verbal claims presented prior to engaging in activities such as exams, tests, oral presentations, among others, and which are used as a plausible excuse for a potential failure (Gupta & Geetika, 2020; Jensen & Deemer, 2020).

Self-handicapping strategies involve early excuses, intentionally created to circumvent the link between ability and performance and protect or increase perceived competence, self-esteem, and self-worth. The obstacle created can be internal (for ex., malaise) or external (for ex., I studied the wrong content), with the main goal to separate a potential failure from personal capacity. In situations where failure is seen as a potential outcome of an achievement, the student may shift an attribution to his own ability, such as “I failed because I’m dumb,” which would cause him/her shame or humiliation to one such as “I failed because I had insomnia last night,” something that is uncontrollable, but which will not cause discomfort in front of other people. In addition, if the student manages to do well in the activity, his ability will be highlighted. In fact, the impetus for handicapping refers to the student’s uncertainty of his ability, which justifies the various strategies for the student to protect his identity or academic “self.” (Coudevylle et al., 2020; Gupta & Geetika, 2020; Jensen & Deemer, 2020; Schwinger et al., 2014; Thompson & Richardson, 2001; Urdan & Midgley, 2001).

Jones and Berglas (1978) were the first to conduct studies in this field, describing self-handicapping as a set of behaviors or claims intended to protect own self-image. More precisely, self-handicapping refers to choices and attitudes adopted to minimize blame for failure and increase the value of success. Until early 1990s, researchers who studied this subject were guided by personality theories and investigations were conducted in a laboratory environment. Covington (1992), Garcia and Pintrich (1993), and Midgley and Urdan (1995) are among the first scholars interested in understanding how self-handicapping strategies are used in the educational context, considering the cognitive, motivational, and interpersonal nature of this phenomenon. Low performance, pessimistic perception of performance, and low self-esteem are plausible reasons for using self-handicapping strategies, which are more often employed in situations where failure has a negative impact on the person, either by the value of the activity or the causal attribution made by others (Thompson & Richardson, 2001).

Arkin and Baumgardner (1985) and Leary and Shepperd (1986) proposed a distinction between behavioral self-handicapping strategies—that is, building obstacles that actually compromise the performance such as missing classes or not studying for an exam—and self-handicapping claims like anxiety, insomnia or having assumed other tasks besides studying. In the first case, the student compromises his performance so his behaviors are open to observation as they are convincing excuses for failure and may result in punishment or another negative consequence. Among behavioral self-handicaps, procrastination is frequently analyzed in the academic field, defined as the act of delaying or postponing a task or an assignment (Geara et al., 2017; Leondari & Gonida, 2007; Schraw et al., 2007; Steel & Klingsieck, 2016). Claimed self-handicaps, however, are elaborate excuses which do not exclude the possibility of dedicated effort, that is, the student who claimed malaise may have studied for the test but will use this excuse to justify in advance a possible bad grade in the test using an explanation that does not put in doubt his ability. In contrast, a good performance may highlight his ability because, despite claiming he could not study enough, he did well in the test. Accordingly, these possible attributions to test results will not be based on the student’s abilities, which allow the protection of his self-worth.

The results of studies carried out in recent decades show self-handicapping is common in the academic environment, and that it is used by both high- and low-achievement students. These individuals follow a cycle of failure–self-handicapping–failure that results in reduced effort and consequent abandonment of the activity (Coudevylle et al., 2020; Gupta & Geetika, 2020; Midgley et al., 1996). Although the use of self-handicapping strategies is very relevant to understanding factors associated with low academic performance in the educational context, the literature has scarce studies and instruments to measure this variable. Some studies addressing the development of instruments to assess self-handicapping strategies will be described next.

### Instruments assessing self-handicapping strategies: a critical review of the literature

Studies analyzing self-handicapping conducted in recent decades relied mainly on two assessment instruments: the Self-Handicapping Scale (SHS; Jones & Rhodewalt, 1982), consisting of 25 items, and a simplified version of 10 items; and the Academic Self-Handicapping Scale (ASHS; Midgley & Urdan, 1995; Urdan et al., 1998). According to Schwinger et al. (2014), although these two instruments are somehow overlapped, the operationalization of the strategies presented in the items is different. Midgley and Urdan (1995) consider important that the instrument items address three aspects: self-handicapping behaviors, reasons that justify such behaviors, and chronology, i.e., the excuse prior to the failure in order to later disregard the student’s ability as a possible attribution. For example, the item “Some students put off studying until the last minute, then, if they do not do well they can say this is the reason. How true is this for you?” Differently, the items of the Self-Handicapping Scale (SHS; Jones & Rhodewalt, 1982) address behaviors with potential self-handicapping, not questioning the reasons for or a priori chronology in relation to the assessment situation (for instance, “ I usually postpone things until the last minute”). Some studies focused on the validation of these two scales are discussed next.

### Self-handicapping Scale (SHS; Jones & Rhodewalt, 1982)

The original instrument of 25 items on a 6-point Likert scale was designed to identify self-handicapping trends as a personality trait. Few studies have attempted to understand its factors and the results are controversial regarding its dimensionality. Data from Rhodewalt et al. (1984) indicated a consistency index (Cronbach’s alpha) of 0.78 and test-retest reliability of 0.74. Strube (1986) carried out a principal component analysis using varimax rotation to assess data from a sample of 168 university students. Six factors were identified, explaining 53.1% of the variance; however, only few items were grouped around the factors and had low consistency, with Cronbach’s alpha index of 0.62. A shorter version with 10 items, which excluded the items with low factor loading, had a Cronbach’s alpha of 0.70. Based on these results, several studies have been conducted with the 25-item scale, the reduced version, or with adaptations to different contexts or educational levels. Table 1 lists the studies that analyzed the instrument, with samples of university students and a validity index.

**Table 1:** Studies with evidence of self-handicapping scale validity

	Authors (year)	Sample	Method	Results
Self-Handicapping Scale (SHS) Jones and Rhodewalt (1982)	Rhodewalt et al. (1984)	27 university students 32 athletes	Internal consistency (Cronbach’s α) Test-retest reliability	α = 0.78 r = 0.74
	Strube (1986)	168 university students	Cronbach’s α Extraction of principal components (PCA)	α = 0.62 6 one-dimensional factors 10 items
	Akın (2012)	585 university students	Cronbach’s α Test-retest EFA AFC	0.90 0.94 1 factor Good fit
	Clarke and MacCann (2016)	484 university students	Parallel analysis EFA (MLE) CFA	2 factors (internal and external) Acceptable fit for 13 items
	Akar et al. (2018). Version: Akın (2012)	350 university students	Cronbach’s α CFA	0.97 Acceptable fit for 25 items
	Barutçu Yıldırım and Demir (2019) Version: Akın (2012)	801 university students	Cronbach’s α	0.74
	Jensen and Deemer (2020)	946 university students	Cronbach’s α (2 factors )	Internal 0.76 External 0.72
	Şahin and Çoban (2020) Version: Akın (2012)	981 university students	Cronbach’s α CFA	0.60 Good fit for 1 factor
	Karami et al. (2020)	360 university students	Cronbach’s α	0.80

The Turkish version of the scale developed by Akin (2012) had its psychometric properties obtained through exploratory factor analysis (EFA) and confirmatory factor analysis (CFA), while the reliability of the original scale was based on Cronbach’s alpha. Clarke and Maccann (2016) exceptionally used parallel analysis, EFA and CFA and found two factors named internal and external aspects of self-handicapping.

Gupta and Geetika (2020) developed the Academic Self-Handicapping, based on the model proposed by Jones and Rhodewalt (1982), with items addressing behaviors with potential self-handicapping, without questioning the reasons for or the chronology in relation to the activity. The validation study was conducted with a sample of 330 high school students in India and, based on the literature, 43 assessment items on a 5-point Likert scale were developed and then reviewed by researchers from this field, who maintained 33 items. An exploratory factor analysis showed a two-factor structure, named behavioral self-handicapping and claims, excluding the items with low factor loading. A confirmatory factor analysis showed adequate indices of adjustment to the model, with the final version consisting of 22 items, showing good evidence of validity for that sample of students.

### Academic Self-Handicapping Scale (ASHS; Midgley & Urdan, 1995; Urdan et al., 1998)

This 6-item scale was developed by Midgley and Urdan (1995) in a study with 256 middle school students and showed an internal consistency index of 0.80. Urdan et al. (1998) conducted a study to investigate the evidence of validity and reliability of the scale (with six items) using EFA (maximum likelihood estimate) and CFA in a sample of 682 fifth-grade students. They identified a one-factor structure with good data fit indices. The scale was predominantly used in studies with children, without alteration of the original six items. Few studies have analyzed university students (for instance, Huff et al., 2016; Chen et al.,^ 2018^) and there were no additional psychometric analyses of the scale.

Overall, self-handicapping strategies have been mostly assessed using quantitative instruments that address the same items (with minor adaptations) contained in the Academic Self-Handicapping Scale (ASHS; Midgley & Urdan, 1995; Urdan et al., 1998) and the Self-Handicapping Scale (SHS; Jones & Rhodewalt, 1982). Some of the results of the major studies were based on elementary and high-school samples (Gupta & Geetika, 2020; Midgley & Urdan, 1995; Urdan et al., 1998). Fewer were the studies which examined the available assessment measures regarding its factors. Data from the research based on principal component analysis and varimax rotation among university students (Strube, 1986) and from other investigations did not reach an agreement concerning the numbers of factors and the dimensionality of the construct (Clarke & Maccann, 2016; Gupta & Geetika, 2020; Midgley & Urdan, 1995; Strube, 1986; Urdan et al., 1998). Not many studies employed multi-methods analyses (Clarke & Maccann, 2016; Strube, 1986). Only one study used parallel analysis (Clarke & Maccann, 2016). Moreover, not all of the studies found high reliabilities indexes. Studies were carried out predominantly with American samples, followed by Australian, Indian, and Turkish samples to a much lesser extent (Akin, 2012; Clarke & Maccann, 2016; Gupta & Geetika, 2020). Also, there are no studies and assessment tools developed to measure self-handicapping strategies which were preceded by qualitative analyses, as well as there were no scales for the Brazilian context.

Considering both the aforementioned issues and the problems regarding the assessment of self-handicapping strategies, as well as the importance of this assessment in a valid and reliable way to better understand factors related to low academic achievement in higher education, this paper reports the results of two studies. Study 1 reports data from a qualitative study which preceded and guided the construction of the Scale for Self-Handicapping Strategies (Escala de Estratégias Autoprejudiciais, EEAPREJ) for university students. It also reports data from quantitative analyses carried out to investigate construct validity of the scale and to estimate its initial psychometric properties. Study 2 describes the results of the EEAPREJ assessment using a parallel analysis with minimum rank factor analysis to find a factor solution for the instrument. The analyses attempted to ensure the construct validity of the EEAPREJ results in a larger Brazilian sample of higher education students.

## Method

### Study 1—qualitative and quantitative analyses undertaken for the construction and initial validation of the Self-Handicapping Strategies Scale (EEAPREJ)

As gathering qualitative information about a construct before developing scale items may be valuable to maximize its content and construct validity (Borg & Gall, 1989; Isaac & Michael, 1982), Boruchovitch and Ganda (2009) developed a qualitative instrument which was used for the construction of the items of the Scale for Self-Handicapping Strategies and to assess its content validity analysis. This instrument presents a hypothetical problem situation based on the literature in this field (Stipek, 1993; Urdan, 2004). It is about a student who uses self-handicapping strategies in the classroom and is asked to think of his behavior in the university course and answer four questions—two closed-ended questions and two open-ended questions. More precisely, the student should report whether or not he uses such strategies, which ones he uses, and whether he thinks it is important to reflect on the acts that disturb his learning.

### Participants and procedures

The sample of this initial study was composed of 27 participants, mostly female (92.59%), from a class of 36 students in the 2nd year of Pedagogy course from a public university in the state of São Paulo who were attending to a Psychology Educational class with a self-reflective emphasis on improving students’ attitudes and behaviors toward learning. Participants’ age ranged from 18 to 28 years (*M =* 20.27, *SD =* 2.27).

Students answered to the hypothetical problem situation in the classroom as an assignment in the presence of Boruchovitch and Ganda (2009) who were teaching the Psychology Educational class in a self-reflective approach. The objectives of the data collection were first explained to the students and those who agreed on participating in the research signed an informed consent. All of students got credit for answering the problem situation. Only those who signed the informed consent had their answers examined by the teachers for research purposes.

### Results

Of the total group, 24 students (89.0%) reported using self-handicapping strategies in their learning. Several participants reported two or more self-handicapping strategies they used in their academic life, totaling 40 different answers. Table 2 shows the main self-handicapping strategies mentioned by participants.

**Table 2:** Major self-handicapping strategies of pedagogy students

Self-handicapping strategies	N/%
Distraction in the classroom (do and think about other things, talk)	10/ 25%
Poor time management for school assignments	6/15%
Failure to do the tasks recommended by professors, such as reading and assignments	6/15%
Lack of efforts to do the tasks well	5/12.5%
Failure to attend classes/miss classes or leave the classroom	3/7.5%
Procrastinate	3/7.5%
Report physical and psychic symptoms, such as tiredness, anxiety, and nervousness	3/7.5%
Sleep very little the day before a class or test	1/2.5%
Make excessive efforts or more than necessary	1/2.5%
Engage in many simultaneous activities	1/2.5%
Lack of organization in the study environment	1/2.5%
**Total**	**40/100%**

The main self-handicapping strategies reported by the students, in order of frequency, were distraction in the classroom/do and think about other things, talk (*n* = 10; 25%); poor time management for school assignments (*n* = 6; 15%); failure to do the tasks recommended by professors, such as reading and assignments (*n* = 6; 15%); lack of efforts to do the tasks well (*n* = 5; 12.5%); failure to attend classes/miss classes or leave the classroom (*n* = 3; 7.5%); procrastinate (*n* = 3; 7.5%); report physical and psychic symptoms, such as tiredness, anxiety, and nervousness (*n* = 3; 7.5%); sleep very little the day before a class or test (*n* = 1; 2.5%); make excessive efforts or more than necessary (*n* = 1; 2.5%); engage in many simultaneous activities (*n* = 1; 2.5%); and lack of organization in the study environment (*n* = 1; 2.5%). Of all self-handicapping strategies mentioned by the participants, behavioral self-handicaps were predominant (92.5%) in their answers, and the only claimed self-handicap was “report physical and psychic symptoms” (7.5%).

### Self-handicapping strategies scale: item construction and content analysis

The first version of the scale was developed by the Boruchovitch and Ganda (2009) with 24 items related to the use of self-handicapping strategies in academic situations, in a 4-point Likert scale where 1 means “it has nothing to do with me” and 4 means “it describes me really well.” The total score of the scale may range from 24 to 96 points—the higher the score, the higher the frequency of self-handicapping strategies reported by students.

Item construction was based both on the literature in this field (Jones & Rhodewalt, 1982; Midgley & Urdan, 1995; Urdan et al., 1998) and the content analysis of students’ answers to the hypothetical problem situation described previously. The wording of each item addressed the three important aspects suggested by Midgley and Urdan (1995): self-handicapping behaviors, reasons that justify them, and chronology. A small pilot study was carried out with the same 27 Pedagogy students who first answered the hypothetical situation who confirmed the intelligibility of the items.

The instrument items were then organized into three subscales according to their content. Each item was read by Boruchovitch and Ganda (2009) independently who rated and classified them into one of the three subscales. Agreement between raters was 100%. The first subscale referred to time management issues (for instance: “some students study only the day before the test. If they do not get a good grade, they say they did not have enough time to study the whole material”). The second subscale was about failure to control attention (for instance: “some students use their cell phones during the class. If they do not get a good grade, they say it was because they did not understand the teacher’s explanation”). The third subscale was related to student preparation for an academic activity (for instance: “some students do not prepare well for an oral presentation and then get very nervous at the time of the presentation. If they do not perform well, they say nervousness affected them”). At this point, each of these subscales contained 12, 5, and 6 items, respectively.

### Content validity of the items

Before the scale was applied to a convenience sample of 164 students from the Pedagogy course of Brazilian public universities, these same students responded to the hypothetical problem situation mentioned previously. As the construction of the items was mostly based in the content analyses of responses of a small sample (*n* = 27), the goal at this point was to check whether similar self-handicapping strategies would be reported in larger and more representative sample.

### Participants and procedures

The sample was composed of 164 students from two Brazilian public universities (*n* = 54; 32.93%) from one institution and (*n* = 110; 67.07%) from another. Among the participants, 147 (89.6%) were female and 17 (10.4%) were male students. Regarding the year of the course, 61 (37.2%) were in the second year and 103 (62.8%) in the fourth year. Ages ranged from 18 to 48 years (*M* = 23.76, *SD* = 5.93). Of the total number of participants, 53 (32.3%) were aged from 18 to 20 years, 97 (59.2%) were between 21 and 30 years old, and 14 (8.5%) were between 31 and 48 years old. Only participants who agreed on taking part of the research by signing an informed consent were included in the sample.

Data collection took place in the classrooms and was conducted by Ganda (2011) who presented the objectives of the study to the students, after the study was approved by the Ethics Committee, on days suggested as more convenient by the teachers of the courses. Students who agreed to participate in the research signed the informed consent. Data collection was carried out collectively and took approximately 20 min. Students responded first to the hypothetical problem situation instrument followed by the EEAPREJ scale.

### Results

The results from the hypothetical situation question showed that, although at different frequencies from the first sample, the same strategies were reported by the participants of this study who provided 174 answers. More precisely, in this study (Ganda & Boruchovitch, 2015), participants mentioned the strategies of procrastination (*n* = 54; 31.0%); failure to read the materials recommended by the professor (*n* = 36; 20.7%); time management issues (*n* = 26; 14.9%), attention control issues (*n* = 25; 14.4%); failure to attend classes/miss classes or leave the classroom (*n* = 17; 9.8%); lack of efforts to do the activities well (*n* = 9; 5.2%); and physical and emotional symptoms (*n* = 7; 4.0%). As the same self-handicapping strategies emerged in the content analysis of the responses of a larger sample, the items were considered as representative of self-handicapping strategies of Brazilian university students enrolled in teacher education programs. Overall, similar self-handicapping strategies were also found in the literature (Gupta & Geetika, 2020; Midgley & Urdan, 1995; Urdan et al., 1998).

Furthermore, internal consistency analyses were carried out for the total scale and its subscales. Cronbach’s alpha coefficient was 0.85 for the total scale and 0.80 for the “time management issues” subscale, and acceptable, though lower values (Prieto & Muñiz, 2000) for the subscales “attention control issues” (α = 0.63) and “lack of efforts to do the activities well” (α = 0.62).

### Construct validity studies: exploratory factor analysis

#### Participants and procedures

Given the lower Cronbach’s alpha coefficients in two subscales, the 24-item scale was again applied to 285 university students from teacher education programs of three public universities located in different states in Brazil: one in the state from Paraná (*n* = 121; 42.5%), one in the state of São Paulo (*n* = 110; 38.6%), and one in the state of Minas Gerais (*n* = 54; 19.0%). The participants were predominantly female (*n* = 262; 91.9%) and were in the 2nd (*n* = 139; 48.8%) and 4th year (*n* = 146; 51.2%) of the Pedagogy course. Of all participants, 45 (15.8%) were under 20 years old, 193 (67.7%) were between 20 and 29 years old, and 47 (16.0%5) were over 30 years old. Only participants who agreed on taking part of the research by signing an informed consent were included in the sample. Data collection took place in the classroom by specially trained research assistants with similar procedures as those employed in the sample with 164 university students mentioned previously.

**Fig. 1:**
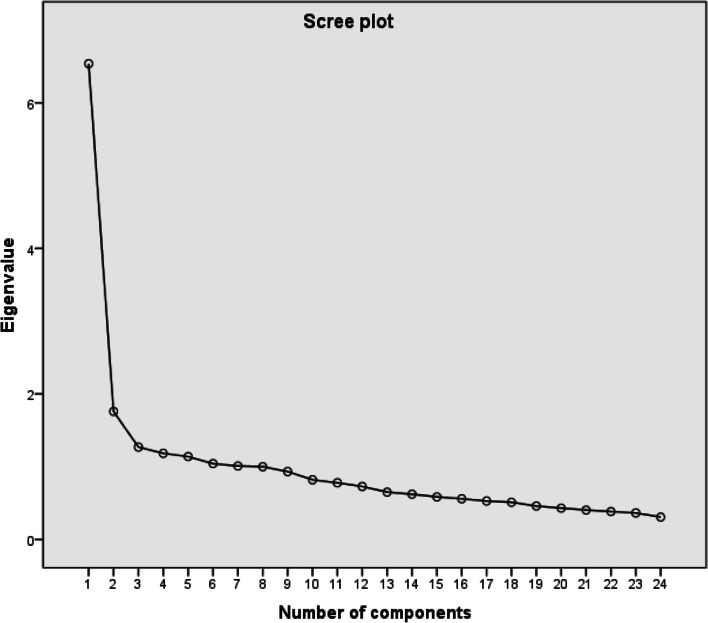
Scree plot test for number of factors of the EEAPREJ scale

An exploratory factor analysis was carried out with the items in the total sample (*N* = 284) to identify the factor structure of the instrument, using the principal component analysis (PCA) and varimax orthogonal rotation. Kaiser’s Measure of Sampling Adequacy: Over-all MSA was 0.8787 and Bartlett test was X2 = 1922.43; df = 276; *p* < .001 indicating that the sample is adequate for factor analysis. By the criterion of selection of factors with eigenvalue greater than 1, 7 factors were obtained, which explained 58.5% of the data variability. Using the scree plot test (Fig. 1), it was decided to set the extraction of 3 factors, which explained 40.41% of the total variability, since from this factor on the curve stabilizes, without further increases in the accumulated percentage of explained variance. Factor 1 explained 27.80%, factor 2 explained 7.33%, and factor 3 explained 5.28%. Table 3 presents the factor loadings after varimax orthogonal rotation.

**Table 3:** Factor composition and items loading after varimax orthogonal rotation

Factors	Loadings	Items
Factor 1	0.7431	20-Some students postpone some important tasks until the deadline set by the professor. If they get a bad grade, they say the assignment was done in the last minute.
	0.6874	21-Some students do not organize their time very well, so they need to stay awake for several nights in a row to do an important assignment. If they get a bad grade, they say they were too sleepy.
	0.6527	23-Some students do not dedicate enough time to an important assignment. If the result is not good, they say they were not engaged.
	0.5706	14-Some students do other things (watch television, listen to music or use the internet) knowing they have little time to do an assignment. If they do not get a good grade, they say the assignment was too long.
	0.5662	1-Some students study only the day before the test. If they do not get a good grade, they say they did not have enough time to study the whole material.
	0.5631	17-Some students do not prepare for a test and then feel very anxious. If they get a bad grade, they blame anxiety.
	0.5460	13-Some students do not read the texts recommended by the professors before the class. If they get a bad grade, they say it was because the texts were too boring.
	0.4662	12-Some students postpone studying and doing academic assignments, and often fail to do them. If they do not do well in the course, they say it was due to lack of time.
	0.4647	22-Some students are focused on non-essential details of an important activity and do not dedicate to its content. If the grade is not what they expected, they say they had no time to do a good assignment.
	0.4535	18-Some students do not pay attention in class, so that if they do not do well in the course, they say that the classes are very boring.
Factor 2	0.7391	4-Some students use their cell phones during the class. If they do not get a good grade, they say it was because they did not understand the teacher’s explanation.
	0.6592	24-Some students read entertainment magazines during classes. If they get a bad grade, they say it was because they did not understand the subject.
	0.657	10–Some students go to parties even when they have an important assignment. If they get a bad grade, they say the proposed assignment was too complex.
	0.5852	5-Some students leave the classroom frequently. If they get a bad grade on the test, they say it was because they missed important content.
	0.5314	11-Some students talk to classmates during the class. If they do not do well in the course, they say that their friends distracted them.
	0.4199	9-Some students always find an excuse, apparently acceptable, to use as an explanation in situations that do poorly in college.
	0.4131	16-Some students miss many classes. If they do poorly in the discipline, they say they did not have access to the content
	0.4130	7-Some students do not prepare well for an oral presentation and so are very nervous at the time of exposure. If they do not perform well, they say nervousness got in the way.
	0.3998	6-Some students go out even when they have an important assignment. If they get a bad grade, they say they had little time to do it.
	0.39811	15- Some students report they have to stay with friends and/or boyfriend/girlfriend. If their assignment is not good, they say they had no time to dedicate to studying.
	0.34124	19- Some students report they have to stay with friends and/or boyfriend/girlfriend. If their assignment is not good, they say they had no time to dedicate to studying.
Factor 3	0.6096	8-Some students intentionally engage in too many activities. If they do not do well in the course, they say they were too busy with other things.
	0.6059	3-Some students do not seriously dedicate themselves to academic activities. If they do not do well in the course, they say they need to help a family member
	0.5442	2-Some students do not study hard and when they do not get a good grade, they say it is not possible because the course has a heavy load.

EEAPREJ is an original instrument developed by Boruchovitch and Ganda in the Brazilian context and in Portuguese language. The items were translated into English for the present publication. Researchers interested in the instrument need to contact authors for proper authorization

The composition of factors took into account only items with loading greater than 0.30 in one of the rotated factors (Kline, 1994). The results showed that, of all 24 original items, 5 items were discarded, 4 of them for loading in a not expected factor (items 3, 7, 16, and 18), and 1 for being too general, not specifying any self-handicapping behavior or claim (item 9). Items 14 and 12 should be disregarded because they obtained high loads in more than 1 factor, but they were allocated to factor 1 due to their content which fitted better in this factor. Overall, according to their content, factor 1 was initially named as Time Management Issues with 9 items (1, 12, 13, 14, 17, 20, 21, 22, 23) and internal consistency of 0.829. Factor 2 was named Attention and Concentration Control Issues with 8 items (4, 5, 6, 10, 11, 15, 19, 24) and internal consistency of 0.751. Factor 3 ended up with 2 items (2, 8) and was difficult to name since their content would probably fit better in factor 1 but they have very low loads in this factor. It also had a very low reliability (alpha of Cronbach = 0.276) as expected facing to be formed by only two items. The alfa of Cronbach of the total scale was 0.860.

Another exploratory factor analysis using the principal component analysis (PCA) and varimax orthogonal rotation was then conducted to check the items in a 2 factor structure. Results showed that the 19 items were organized into 2 factors: factor 1 was named Time Management Issues, consisting of 11 items (1, 2, 8, 12, 13, 14, 17, 20, 21, 22, 23) with internal consistency of 0.826, and factor 2 was named Attention and Concentration Control Issues, containing the same 8 items of the previous analysis (4, 5, 6, 10, 11, 15, 19, 24) with internal consistency of 0.751. Cronbach’s alpha for the full scale was 0.860. These two factors explained 35.13% of the variance (27.80% and 7.33% respectively). However, some problems arose. Items 12, 13, and 14 (factor 1) and items 6 and 15 (factor 2) had loads greater than 0.30 in two factors. These items were allocated to the factors in which their content fitted better and they loaded higher. Items 12, 13, and 14 remained in factor 1 while items 6 and 15 in factor 2. Moreover, item 2 had a load of 0.292 in factor 1. The decision was to keep this item due to its content and better examine it in future studies. Overall, most of the items which loaded in factor 1 were related to time management problems and the ones loaded in factor 2 were associated with attention and concentration problems, but not all of them.

Though the EEAPREJ differs from other available instruments for the assessment of self-handicapping strategies, due to its qualitative origin of its items which were constructed based on the self-report of university students about their experience in employing self-handicapping strategies in academic situations, the validity evidence resulting from the internal consistency index analysis of the subscales (measured by Cronbach’s alpha) and scale dimensionality by the PCA, as well as the low percentage of variance explained by the factors in the Brazilian sample, were not sufficient to confirm the instrument accuracy and validity.

The problems that emerged in previous exploratory factor analyses of EEAPREJ coupled with the fact that the literature available is also controversial about the dimensions of this construct as some authors found six factors while others found one or two motivated a deeper investigation of this construct. Accordingly, further analysis of the items, using more recently statistical procedures recommended in the literature (Asún et al., 2016; Damásio, 2012; Hayton et al., 2004; Holgado–Tello et al., 2010), were conducted with the EEAPREJ in Brazil, using its 19-item version and will be reported in study 2.

### Study 2—exploratory factor analysis using parallel analysis, based on minimum rank factor analysis method and the matrix of polychoric correlations

In study 2, we sought to find out the factor structure underlying the observable items of the EEAPREJ using more recent statistical analyses, when compared to those employed in study 1. Although exploratory analyzes had already been carried out in study 1, PCA was used to estimate components. PCA is a technique that may have led to groupings of items inconsistently with the theory underlying the construct, in addition to low factor loadings between the item and the retained factor or factors with a reduced number of items with low factor loading. Thus, to justify the reasons a new EFA was carried out, some brief considerations about the analyses will be made next.

EFA is constantly evolving due to the use of new software and the potential of new generations of computers, which allow for greater speed in the execution of analyzes and the implementation of new techniques. PCA, even with limitations for use in research in the field of psychology, is historically the most used and still appears in many current studies. In SPSS software, it appears as one of the methods available for EFA, leading researchers to choose this option. In fact, EFA and CPA are different techniques, although commonly used in research to analyze the factor structure of instruments. Taking into account that the variance of each observable variable is composed of specific variance (of the variable itself), common variance (shared by all items of the factor or component), and error variance (part of the item not explained by the component or factor), PCA is based on the linear correlation between the observable variables (items), joining the common and specific variances, inflating the factor loadings, and commonalities. It is considered as a formative model, that is, the items generate the component, and its use is pertinent to areas of administration, economics, or sociology. For example, from the collection of categorical data such as sex, age, salary range, education level, neighborhood of residence, a socio-economic component can be generated (Coltman et al., 2008; Damásio, 2012; Fabrigar et al., 1999; Fokkema & Greiff, 2017; Joliffe & Morgan, 1992).

According to Damásio (2012), Hernandez et al. (2017), and Joliffe and Morgan (1992), the objective of the EFA is to discover the underlying structure of a data matrix, as well as to specify the nature and number of latent variables (factors) that best represent a set of observable variables (items). The EFA derives from the reflexive model, whose objective is to discover the structure of latent constructs in the instrument’s set of items, with the explained variance being the portion of common variance extracted from the analyzed data set. In addition, factor loadings that explain between 30 and 40% of the common variance among the factor items would consider a large part of unexplained variance, and it is advisable to retain only items with a loading greater than 0.50 (Costello & Osborne, 2005; Damasio, 2012).

In addition to the limitations of PCA as a method of item reduction in psychology studies, according to Cortina (1993), Damásio (2012), and Sijtsma (2009), Cronbach’s alpha has several limitations, because it considers that all items have the same importance for the factor and have a linear correlation with each other. Furthermore, the consistency assessed refers to the level of interrelationship between the variables, and not to homogeneity, which refers to the construct dimensionality.

### Participants

The sample was a convenience sample and consisted of 834 university students, from various undergraduate courses such as Pedagogy (*n* = 601; 67.99%), History (*n* = 23; 2.60%), Geography (*n* = 30; 3.39%), Mathematics (*n* = 27; 3.05%), Language (*n* = 56;6.33%), Biology (*n* = 15;1.70%) Sociology (*n* = 17;1.92 %), Nursing (*n* = 9 ;1.02%), Visual Arts (*n* = 1; 0.11%), Physical Education (*n* = 55; 6.22%), Physics (*n* = 47; 5.32%), Chemistry (*n* = 1; 0.11%), and Music (*n* = 2; 0.23%), from different public (*n* = 719; 81.33%) and private (*n* = 165; 18.66%) Brazilian universities. More precisely, 407 (46.04%) of the students were from São Paulo, 129 (14.59%) from Paraná, 102 (11.54%) from Ceará, 77 (8.71%) from Bahia, 76 (8.59%) from Goias, 55 (6.22%) from Minas Gerais, and 38 (4.30%) from Mato Grosso do Sul. Their age ranged between 17 and 57 years, mean age 24.40, most of them were female (81.1%), attending the 1st to the 6th year of their courses. The majority of the sample attended classes at night (*n* = 450; 51.14%), followed by in the morning (*n* = 236; 26.82%), the whole day (*n* = 180; 20.45%), in the afternoon (*n* = 14; 1.59%) and 4 (0.45%) missed this question.

### Procedures

The study is part of a larger research project, submitted to and approved by the State University of Campinas Ethics Committee, under n° CAAE: 19230913.9.0000.5404. Authorized by the coordinators of the university courses, the students, after signing an informed consent form (ICF) in duplicate, answered the scale in the classroom on days previously agreed by the professors in the presence of trained researchers. The researchers explained the study objectives and highlighted the importance of students answering the questions sincerely, since there were no right or wrong answers, and they were available to answer any question. Data collection regarding self-handicapping scale took approximately 15 min, without any problems or disruptions.

### Data analysis

Data related to all 834 students who answered the EEAPREJ were exported to FACTOR 11.02.04 to perform an exploratory factor analysis (EFA) using parallel analysis (Pa), based on minimum rank factor analysis (MRFA), and Robust Promin rotation, which allows an interpretation of the common variance extracted from the analyzed dataset, minimizing the common residual variance. The parallel analysis method has been widely used in international studies (Clarke & MacCann, 2016, for instance, in the assessment of the Self-Handicapping Scale), providing accurate results while searching for a factor solution, which are essential for studies that use questionnaires with statements on Likert-type scales. The method compares eigenvalues (portion of total explained variance) extracted from the data matrix with the mean of eigenvalues (portion of total variance) from the simulation of 500 sets of polychoric correlation matrices, equal in number of variables, cases observed, type of covariance matrix, and factor extraction method, with the random permutation of observed values (Asún et al., 2016; Damásio, 2012; Timmerman & Lorenzo-Seva, 2011).

The analysis of score distribution among the items showed excess asymmetry in some items (general values between 0.62 and 3.64); for example, item 7 “Some students go to parties even when they have an important assignment. If they get a bad grade, they say the proposed assignment was too complex” (2.91); or excess kurtosis (general values between − 0.40 and 13.54); for example, item 19 “Some students read entertainment magazines during classes. If they get a bad grade, they say it was because they did not understand the subject” (13.94). Specifically in this item, 736 participants answered option 1 (it has nothing to do with me) and 69 selected option 2 (it has little to do with me). According to Mardia (1970), a violation of the assumption of multivariate normality of data, with asymmetry coefficients 83.62 (*p* = 1.00) and kurtosis 647,704 (0.0000), justified the conduction of a robust exploratory factor analysis based on the matrix of polychoric correlations of the scale items (Table 4).

**Table 4:** Matrix of polychoric correlations of all 19 items of the EEAPREJ scale

V	1	2	3	4	5	6	7	8	9	10	11	12	13	14	15	16	17	18	19
1	1																		
2	0.41	1																	
3	0.34	0.37	1																
4	0.40	0.40	0.67	1															
5	0.41	0.41	0.57	0.65	1														
6	0.23	0.34	0.33	0.45	0.51	1													
7	0.31	0.41	0.60	0.61	0.63	0.57	1												
8	0.27	0.32	0.56	0.48	0.48	0.38	0.63	1											
9	0.45	0.38	0.35	0.43	0.50	0.43	0.53	0.48	1										
10	0.37	0.37	0.44	0.40	0.45	0.37	0.46	0.43	0.56	1									
11	0.42	0.39	0.45	0.39	0.50	0.41	0.51	0.47	0.59	0.57	1								
12	0.32	0.29	0.46	0.51	0.59	0.47	0.56	0.45	0.54	0.50	0.66	1							
13	0.25	0.28	0.35	0.35	0.35	0.37	0.44	0.42	0.42	0.41	0.44	0.35	1						
14	0.08	0.28	0.29	0.33	0.32	0.33	0.46	0.42	0.31	0.27	0.31	0.33	0.39	1					
15	0.37	0.25	0.25	0.29	0.42	0.39	0.36	0.36	0.61	0.50	0.55	0.45	0.35	0.42	1				
16	0.39	0.36	0.30	0.34	0.45	0.41	0.42	0.39	0.53	0.44	0.49	0.43	0.41	0.38	0.59	1			
17	0.27	0.37	0.24	0.32	0.39	0.47	0.45	0.31	0.46	0.42	0.45	0.43	0.42	0.40	0.47	0.57	1		
18	0.39	0.31	0.19	0.32	0.35	0.31	0.32	0.28	0.53	0.41	0.50	0.45	0.36	0.36	0.61	0.53	0.50	1	
19	0.24	0.39	0.53	0.55	0.59	0.43	0.63	0.61	0.38	0.47	0.51	0.54	0.45	0.46	0.38	0.32	0.43	0.33	1

*V* variables

The Kaiser-Meyer-Olkin (KMO) test had a value of 0.93, which is a very good score. According to Damásio (2012), the test, also known as the sampling adequacy index, indicates the proportion of variance of the items that can supposedly be explained by a latent variable. Bartlett’s test of sphericity identifies the extent to which the (co)variance matrix is similar to an identity matrix and the overall significance of all correlations in a data matrix, *p* values < 0.05 indicating that the matrix is factorizable. In our study, the value of 8862.2 (171) was obtained (*p* < 0.001), with both tests indicating the data adequacy for the factor analysis.

The EFA showed a factor solution of 3 factors with eigenvalues > 1.0; however, the parallel analysis based on MRFA (Timmerman & Lorenzo-Seva, 2011) recommended the retention of only one factor (eigenvalue 8.49), explaining 61.9% of the common variance. Table 4 shows the percentages of variance of current data, random mean and random percentile.

As indicated in Table 5, the hypothesis that the EEAPREJ contained three dimensions (time management issues, attention control issues, and failure to perform an assignment) was not supported by the EFA, indicating a dimensional solution of only one factor.

**Table 5:** Parallel analysis based on minimum rank factor analysis (MRFA)

Variables	% of variance		
	Current data	Random mean	95 percentile random order
1	49.96^a^	10.51	11.83
2	8.82	9.74	10.79
3	5.74	9.12	10.02

Number of 500 random matrices of polychoric correlation. Method: raw data permutation

^a^Number of factors recommended when 95th percentile is considered

The items of the EEAPREJ scale showed high factor loadings, ranging from 0.51 to 0.77, as well as satisfactory communalities, with values from 0.54 to 0.93 (Table 6).

**Table 6:** Factor loadings, means and standard deviations, asymmetries, kurtosis and commonalities of EEAPREJ items

Item	F	M	SD	As	Ku	h2
1. Some students study only the day before the test. If they do not get a good grade, they say they did not have enough time to study the whole material.	0.51	1.95	0.84	0.68	− 0.07	0.55
2. Some students do not study hard and when they do not get a good grade, they say it is not possible because it is a heavy course load.	0.54	1.55	0.73	1.25	1.12	0.61
3. Some students use their cell phones during the class. If they do not get a good grade, they say it was because they did not understand the teacher’s explanation.	0.65	1.41	0.72	1.82	2.82	0.84
4. Some students leave the classroom frequently. If they get a bad grade on the test, they say it was because they missed important content.	0.69	1.33	0.67	2.18	4.31	0.78
5. Some students go out even when they have an important assignment. If they get a bad grade, they say they had little time to do it.	0.75	1.37	0.70	1.96	3.28	0.75
6. Some students intentionally engage in too many activities. If they do not do well in the course, they say they were too busy with other things.	0.63	1.57	0.81	1.35	1.07	0.66
7. Some students go to parties even when they have an important assignment. If they get a bad grade, they say the proposed assignment was too complex.	0.77	1.23	0.59	2.91	8.64	0.76
8. Some students talk to classmates during the class. If they do not do well in the course, they say that their friends distracted them.	0.68	1.35	0.67	2.08	4.17	0.75
9. Some students postpone studying and doing academic assignments, and often fail to do them. If they do not do well in the course, they say it was due to lack of time.	0.74	1.62	0.83	1.21	0.65	0.77
10. Some students do not read the texts recommended by the professors before the class. If they get a bad grade, they say it was because the texts were too boring.	0.67	1.77	0.83	0.88	− 0.03	0.57
11. Some students do other things (watch television, listen to music or use the internet) knowing they have little time to do an assignment. If they do not get a good grade, they say the assignment was too long.	0.74	1.67	0.86	1.01	0.15	0.72
12. Some students report they have to stay with friends and/or boyfriend/girlfriend. If their assignment is not good, they say they had no time to dedicate to studying.	0.73	1.42	0.73	1.73	2.36	0.81
13. Some students do not prepare for a test and then feel very anxious. If they get a bad grade, they blame anxiety.	0.58	1.69	0.85	1.07	0.35	0.54
14. Some students study the wrong content for the test. If they get a bad grade, they say that is the reason.	0.54	1.31	0.64	2.26	5.00	0.69
15. Some students postpone some important tasks until the deadline set by the professor. If they get a bad grade, they say the assignment was done in the last minute.	0.68	1.85	0.94	0.83	− 0.33	0.92
16. Some students do not organize their time very well, so they need to stay awake for several nights in a row to do an important assignment. If they get a bad grade, they say they were too sleepy.	0.69	1.69	0.91	1.13	0.20	0.69
17. Some students are focused on non-essential details of an important activity and do not dedicate to its content. If the grade is not what they expected, they say they had no time to do a good assignment.	0.64	1.59	0.81	1.23	0.70	0.69
18. Some students do not dedicate enough time to an important assignment. If the result is not good, they say they were not engaged.	0.60	1.84	0.92	0.79	− 0.40	0.61
19. Some students read entertainment magazines during classes. If they get a bad grade, they say it was because they did not understand the subject.	0.73	1.17	0.53	3.64	13.94	0.93
Mean (standard deviation)	29.45 (8.75)					

(*F* factor, *M* mean, *SD* standard deviation, *As* asymmetries, *Kur* kurtosis, *h2* communalities)

EEAPREJ is an original instrument developed by Boruchovitch and Ganda in the Brazilian context and in Portuguese language. The items were translated into English for the present publication. Researchers interested in the instrument need to contact authors for proper authorization

## Discussion

The EEAPREJ scale was designed to assess the use of self-handicapping strategies in the academic context by Brazilian university students from different majors of several universities. To analyze its psychometric properties of validity and reliability was the main objective of the present study. The scale development process described in study 1 began with the self-report of 27 students about their experience of using these strategies in their academic activities. The content of their answers was taken into account while creating the 24 items of the EEAPREJ scale. Items were considered representative of actual self-handicapping behaviors and claims university students tend to engage in.

The first analyses of the EEAPREJ reported in study 1 of this article assessing its validity were based on the internal consistency indices (Cronbach’s alpha), factor dimensionality, and principal component analysis (PCA). Results revealed that despite the positive aspects of data fit, they did not generate sufficient initial evidence of validity, since problems did emerge mostly related to the fact that some items loaded high in two factors. In fact, most psychometric studies using scales to assess the use of self-handicapping strategies (SHS; Jones & Rhodewalt, 1982 and ASHS; Midgley & Urdan, 1995; Urdan et al., 1998) were also based on the internal consistency indices (Cronbach’s alpha) and principal component analysis (PCA), yielding controversial results regarding the dimensionality of the construct, name, and number of the factors as well as the content validity of the items.

In study 2, data from 834 university students were analyzed and the results from a parallel analysis showed a one-dimensional structure of the scale. The analyses carried out in study 2 used statistical techniques recommended by current psychometric experts (Asún et al., 2016; Costello & Osborne, 2005; Damásio, 2012; Ferrando & Anguiano-Carrasco, 2010; Hernandez et al., 2017).

The score distribution among the EEAPREJ items showed excess of asymmetry and kurtosis in some items, justifying the use of polychoric matrix. In consonance, using a parallel analysis to study dimensionality seemed more appropriate and provided a higher level of accuracy (Damásio, 2012; Hayton et al., 2004; Hernandez et al., 2017; Matos & Rodrigues, 2019). The one dimension structure found in study 2 was also reported in another study with basic education students using different statistical procedures (Urdan et al., 1998).

The analyzes carried out in study 2 did not confirm the hypothetical dimensions that led to the elaboration of the items, suggesting that the construct is one-dimensional and sensitive to identify self-handicapping strategies as a single set.

Although this paper is not focused on discussing statistical analysis, the differences in results obtained with the EEAPREJ in study 1 (with samples of Brazilian students) and study 2 (with 834 Brazilian students) can be explained as a result of the different methods used in the analyses.

Study 2 also provided evidence that the scale has good psychometric properties, but, despite this evidence, the present study has several limitations that should be overcame by further research. The sample in which the item construction was based was indeed small. Content validity of the items was estimated based on the reapplication of the hypothetical situation to larger sample. Items should have been exposed to a group of expert judges to better assess their content validity. The sample in which the exploratory factor analyses were carried out, though not small (ratio 11 students per item), was not representative of the university student. Additionally, although the sample of the study 2 was large (ratio 43.89 students per item), it was also not representative of the university students, since it was heavily based on students enrolled in teacher education programs, as well. Moreover, variables such as major, age, gender, semester of the course, among others, could have affected the report of use of self-handicapping strategies and were not balanced in the sample.

As individuals tend to minimize their faults and maximize their virtues (Hopkins, Hopkins et al., 1990), and probably this is the general principle behind the employment of self-handicapping strategies, it is advisable that observational and focal group studies be conducted as complimentary to the use of the scale. Additionally, it is suggested that the EEAPREJ scale be applied in a sample of university students in order to carry out a confirmatory factor analysis to certify the choice of indicators of the hypothetical construct. The strategy for indicating the fit of the model to the sample data will also reveal the adequacy or not of items, for example, those with excessive kurtosis.

## Conclusion

The availability of valid instruments that can be used in research reflects the comprehension of investigated constructs and the feasibility of intercultural studies analyzing different samples. It is important for the theory that assumes the existence of the phenomenon, enabling its acceptance, alteration, or even rejection. In the case of self-handicapping strategies, although proposed almost 40 years ago, gaps and doubts are still observed in their measurements.

Though evidence of the sound psychometric properties of EEAPREJ has been provided, the consolidated use of this scale in research will certainly require further studies in different contexts, involving other variables and constructs. Further research needs to direct efforts toward analyzing how self-handicapping strategies are related to demographic variables (age, gender, ethnicity), psychological variables (self-efficacy, self-esteem, causal attribution, academic anxiety, to name a few), and academic life variables (adaptation, learning, academic performance). Studies with more heterogeneous samples that can confirm the psychometric properties and explore other types of validity of the EEAPREJ scale are equally important.

Moreover, it is essential to identify students’ actions that enhance and/or hinder learning to strengthen or avoid them in order to prevent problems of adaptation and drop out in higher education. The EEAPREJ scale can be a useful measure to assess university student self-handicapping behaviors. Its use in higher education will certainly be promising for diagnosis and implementation of actions to improve learning and the academic performance at this educational level. The EEAPREJ scale may be also useful to researchers from other countries, inspiring new studies of translation, adaptation and validation, contributing to the advancement of cross-cultural research in assessment measures.
